# Stress and DNA Methylation of Blood Leukocytes among Pregnant Latina Women

**DOI:** 10.3390/epigenomes7040027

**Published:** 2023-11-01

**Authors:** Veronica Barcelona, Sameera Abuaish, Seonjoo Lee, Sarah Harkins, Ashlie Butler, Benjamin Tycko, Andrea A. Baccarelli, Kate Walsh, Catherine E. Monk

**Affiliations:** 1 Columbia University School of Nursing, New York, NY 10032, USA; seh2238@cumc.columbia.edu; 2 Department of Basic Sciences, College of Medicine, Princess Nourah bint Abdulrahman University, P.O. Box 84428, Riyadh 11671, Saudi Arabia; syabuaish@pnu.edu.sa; 3 Mental Health Data Science, New York State Psychiatric Institute, New York, NY 10032, USA; seonjoo.lee@nyspi.columbia.edu; 4 Department of Biostatistics, Columbia University Mailman School of Public Health, New York, NY 10032, USA; 5 Department of Psychiatry, Columbia University Vagelos College of Physicians and Surgeons, New York, NY 10032, USA; cem31@cumc.columbia.edu; 6 Department of Obstetrics and Gynecology, Columbia University Vagelos College of Physicians and Surgeons, New York, NY 10032, USA; adb2167@cumc.columbia.edu; 7 Hackensack Meridian Health Center for Discovery and Innovation, Nutley, NJ 07110, USA; benjamintycko@gmail.com; 8 Department of Environmental Health Sciences, Columbia University Mailman School of Public Health, New York, NY 10032, USA; ab4303@cumc.columbia.edu; 9 Department of Psychology, University of Wisconsin-Madison, Madison, WI 53706, USA; klwalsh2@wisc.edu; 10 New York State Psychiatric Institute, New York, NY 10032, USA

**Keywords:** epigenetics, pregnancy, stress, Hispanic, Latino

## Abstract

Latinas experience physical and psychological stressors in pregnancy leading to increased morbidity and higher risk for adverse birth outcomes. Epigenetic changes, including DNA methylation (DNAm), have been proposed as markers to create more refined risk stratification, yet few of these studies have examined these changes in Latinas. We conducted a secondary analysis of stored blood leukocytes of Latina women (n = 58) enrolled in a larger National Institutes of Health funded R01 project (2011–2016). We examined DNAm on eight candidate stress genes to compare physically and psychologically stressed participants to healthy (low stress) participants. We found unique CpGs that were differentially methylated in stressed women early- and mid-pregnancy compared to the healthy group, though none remained significant after FDR correction. Both physical and psychological stress were associated with hypomethylation at two consecutive CpG sites on NR3C1 in early pregnancy and one CpG site on NR3C1 in mid-pregnancy before adjustment. Stress was also associated with hypomethylation at two CpG sites on FKBP5 in early and mid-pregnancy but were no longer significant after FDR adjustment. Though we did not find statistically significant differences in DNAm during pregnancy between stressed and healthy women in this sample, signals were consistent with previous findings. Future work in larger samples should further examine the associations between stress and DNAm in pregnancy as this mechanism may explain underlying perinatal health inequities.

## 1. Introduction

Latinos comprise the largest ethnic minority group in the United States (U.S.) [1], with the highest fertility rates second only to Alaska Natives [2]. Latina women are at increased risk of experiencing stressors in pregnancy [3] due to structural and social barriers such as lack of access to insurance and health care services [4,5]. Psychological stressors of acculturation and discrimination in Latina women have been associated with an increased risk of adverse birth outcomes, including preterm birth and low birth weight [6]. Latina women are also more likely to experience physical stressors such as food insecurity, poor diet quality, and physical inactivity than White women, and have higher rates of subsequent obesity [7]. Pregnant Latina women are also at higher risk of severe maternal morbidity [8] leading to preterm birth and an increased risk of gestational diabetes [9], often resulting in the development of Type 2 diabetes after pregnancy. Hypertension is also a significant problem among Latino adults, with a steadily increasing prevalence [10]. 

Though most studies examining epigenetics of stress in pregnancy have focused on infant outcomes [11], recent studies have begun to examine how psychological and physical stressors interact to produce poor maternal health [12], likely mediated through the Hypothalamic-Pituitary-Adrenal (HPA) axis [13]. The molecular mechanisms underlying the HPA regulation of stress pathways in pregnancy, however, are not completely understood. Further, there is marked heterogeneity in pregnancy-related morbidity and adverse birth outcomes among Latino subgroups [14], highlighting the need for studies examining within-group differences in a well characterized study sample. Improved knowledge of how stressors are associated with Latina pregnancy health is crucial to (1) create more refined risk stratification and screening approaches in pregnancy and (2) develop translational precision health treatment approaches to reduce preventable maternal morbidity in pregnancy. 

The molecular mechanisms underlying the pathways from stressors to poor perinatal health are thought to involve epigenetic mechanisms such as DNA methylation (DNAm) as it may mediate the relationship between stress and poor health outcomes via gene expression [15]. DNAm is the most frequently studied epigenetic mechanism that involves the addition or removal of a methyl group to the 5′ cytosine ring at cytosine-phosphate-guanine (CpG) dinucleotide bases. Often, DNAm reduces or eliminates gene expression. Increasing attention has been paid to the study of perinatal epigenetics, and to changes in gene expression that do not alter the DNA sequence. 

Previous research has examined stress during pregnancy and methylation of glucocorticoid pathway and stress genes, though most of these studies have conducted DNAm analyses in the immediate postpartum period [16,17,18,19,20,21]. In a sample of pregnant women (N = 83) from Belgium, psychosocial stress and cortisol was measured at each trimester, and pregnancy-related anxiety was found to be associated with *NR3C1* methylation in cord blood [16]. A case-control study of postpartum Mexican women (N = 40) compared women with preeclampsia and normotensive women and found differential methylation patterns in promotor regions of genes, including *NR3C1* among women with preeclampsia, suggesting a regulatory role in response to stress in pregnancy [18]. Another study examined chronic and war-related stress among pregnant women (n = 24) from the Democratic Republic of Congo and methylation of HPA genes at birth. That group reported differential methylation of *CRH*, *CRHBP*, *NR3C1*, and *FKBP5* for women with higher levels of reported stress [19].

Only two studies to date have examined psychological stressors in pregnancy and epigenetics among Latinas during pregnancy [20,21]. Santos et al., reported that DNAm of *NR3C1* was correlated with psychological distress in a cross-sectional analysis of n = 150 Latinas [20]. In that study, racial discrimination was the measured stressor and DNAm was assessed in blood between 24–32 weeks gestation. They reported that hypomethylation of *NR3C1* was associated with stressors such as everyday discrimination. In the other study, Santos’ group also studied experiences of discrimination and DNAm of stress related genes in Latina mothers in a cross-sectional analysis of blood DNAm at 24–32 weeks gestation (n = 147) [21]. They found that discrimination was associated with hypomethylation of *NR3C1* and *FKBP5* in mid-pregnancy.

Progress towards understanding the relationship between stress in pregnancy and epigenetic changes has been hampered by cross-sectional study designs, lack of integration of epigenomic markers with traditional measures, and limited inclusion of high-risk and marginalized populations within the United States in research studies. Knowledge of the timing of stress induced DNAm changes may inform future screening using DNAm as a biomarker. In addition, within-group comparisons are needed for the study of poor maternal health outcomes as unique stressors associated with immigration, language barriers, or cultural factors may not be shared by other racial and ethnic groups [22]. Concentration on within-group analyses among high stress risk groups including a comparison group of healthy women provides more heterogeneity in the sample and increases the chances of finding differences where they exist.

Few studies have examined methylation of stress-related genes in pregnancy, though some genes associated with stress have been studied [20,21]. Two genes that are known to be involved in stress response regulation are *NR3C1* and *FKBP5*. Methylation at specific CpG sites on *NR3C1*, a glucocorticoid receptor gene [23], has been associated with adversity and stress in several studies [24]. Stress and adversity have also been associated with demethylation of *FKBP5*, contributing to increased recovery time after stress exposures, glucocorticoid resistance, and higher cortisol levels [25]. *BDNF* is involved in HPA function and glucocorticoid interactions associated with stress measures [26]. The remaining selected genes (*HSD11B2*, *SLC6A4*, *CRHR1*, *CRHR2*, and *NR3C2*) are involved in HPA functioning and methylation of these genes has been associated with antenatal maternal stress [27,28]. 

The purpose of this pilot study of Latina pregnant women was to examine DNAm of candidate stress genes (*NR3C1*, *BDNF*, *FKBP5*, *HSD11B2*, *SLC6A4*, *CRHR1*, *CRHR2*, and *NR3C2*), and to compare in DNAm among physically and psychologically stressed and healthy phenotypes at two timepoints in pregnancy. We hypothesized that women in the stressed groups would have different methylation patterns compared to those in the healthy group. We focused on reporting the effect sizes of a list of genes suggested by the literature.

## 2. Results

**Figure 1:**
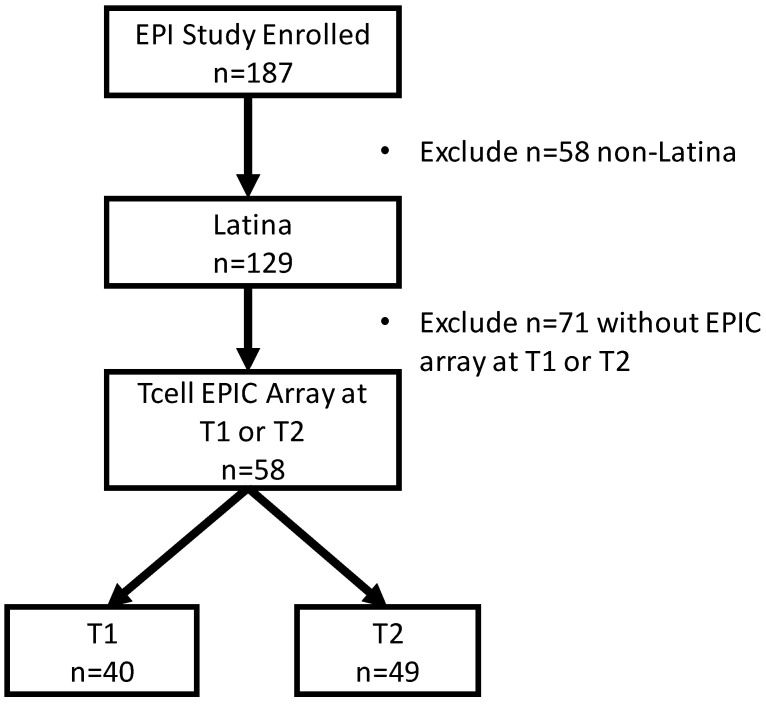
Flow chart of participant inclusion in the present study.

Our study included data from N = 58 Latina participants who had blood samples drawn at T1, T2, or at both the T1 and T2 visits. This resulted in 40 women providing samples at T1 and 49 at T2 (Figure 1). All women self-identified as having Latina ethnicity. The mean maternal age across the sample was 27.1 years, and most were nulliparous, graduated high school (70.2%), and reported incomes less than $50,000 per year (Table 1). There were no differences in gestational age at birth by group (*p* = 0.55). None of the participants reported that they were current smokers, though approximately a third reported having smoked before the pregnancy. 

**Figure 2:**
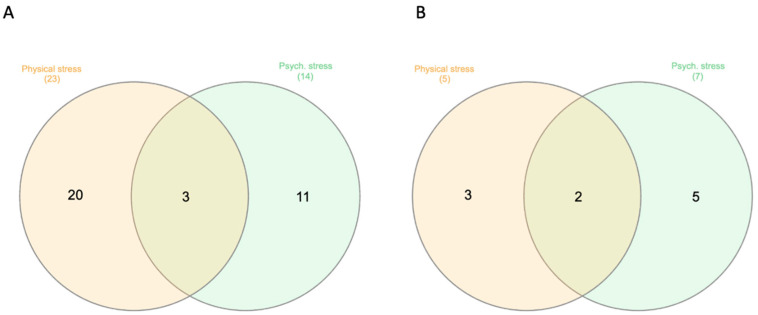
Venn diagram of the number of differentially methylated CpG sites before FDR adjustment in Physically and Psychologically stressed women relative to healthy women at T1 (**A**) and T2 (**B**).

We investigated a total of 409 CpGs in the eight candidate genes chosen for this study (Table 2), and though our findings were consistent in terms of direction between the two stress groups, including two consecutive CpG sites, none passed FDR correction. Before FDR adjustment, raw *p*-values revealed a total of 34 unique CpGs that were differentially methylated in stressed women at T1 relative to healthy women, of which 23 and 14 CpGs differentially methylated in physically and psychologically stressed women, respectively (Figure 2A). In physically stressed women, there were 8 differentially methylated CpGs in *BDNF*, 1 in *CRHR1*, 3 in *CRHR2*, 4 in *FKBP5*, 5 in *NR3C1*, and 2 in *NR3C2*, while there were no differentially methylated CpGs found in *HSD11B2* and *SLC6A4*. Psychologically stressed women had a total of 14 differentially methylated gene across the 8 candidate genes, with 2 differentially methylated CpGs in *BDNF*, 3 in *CRHR1*, 1 in *CRHR2*, 2 in *FKBP5*, 2 in *NR3C1*, 2 in *NR3C2*, 1 *HSD11B2*, and 1 in *SLC6A4.* There were 3 differentially methylated CpG sites in common between physical and psychological stress groups; 2 in *NR3C1* and 1 in *FKBP5* (Figure 2A). However, no sites remained significant after FDR correction. We reported detailed information on percent average methylation, beta values (methylation difference), and standardized effect size (Cohen’s d) in Supplementary Table S1.

At T2 there were 12 differentially methylated CpGs in stressed women relative to healthy women (Figure 2B). Physically stressed women had a total of 5 differentially methylated CpGs with 1 CpG in *CRHR1*, *CRHR2*, and *FKBP5*, along with 2 CpGs in *NR3C1*. There were no differences in *BDNF*, *NR3C2*, *HSD11B2*, and *SLC6A4.* In psychologically stressed women, there were a total of 7 differentially methylated genes with 1 CpG in *CRHR1*, *CRHR2*, *FKBP5*, and *NR3C2*, in addition to 3 CpGs in *NR3C1.* There were 2 differentially methylated CpG sites in common between physical and psychological stress groups belonging to *NR3C1* and *FKBP5* (Figure 2B). However, after FDR correction, no sites were statistically significant. 

**Figure 3:**
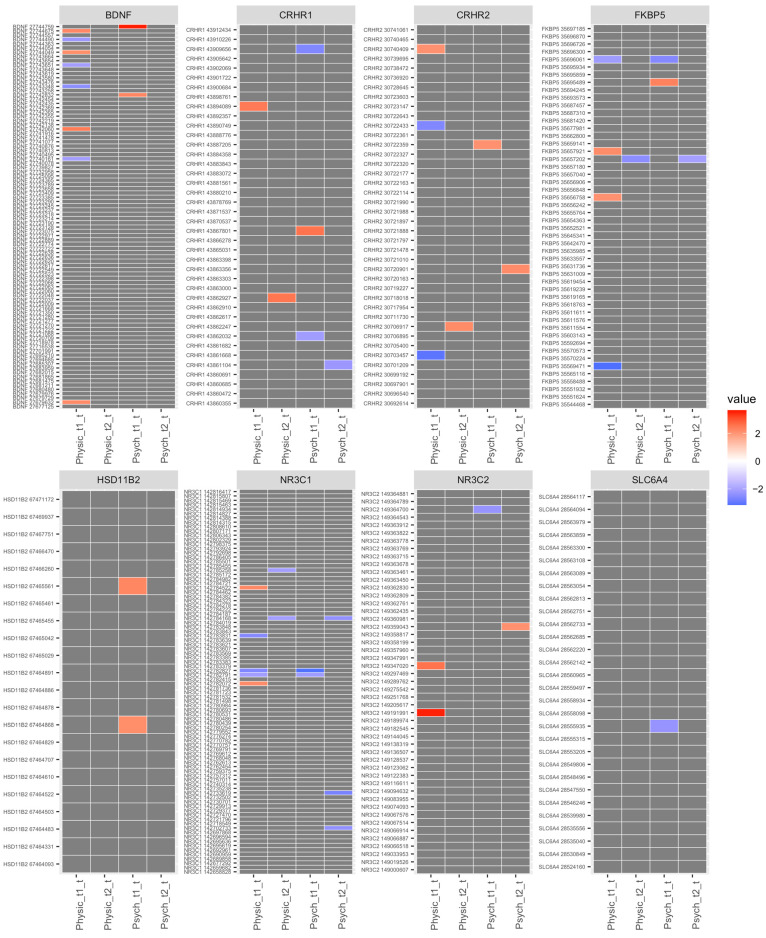
CpG sites for candidate genes for each stress group by genomic position and time point. Significantly differentiated methylated sites (*p*-value < 0.05) are represented by their t-value with red indicating hypermethylation (darker red indicating more hypermethylated) and blue hypomethylation (darker blue indicating more hypomethylated) in stress groups relative to the healthy group.

**Figure 4:**
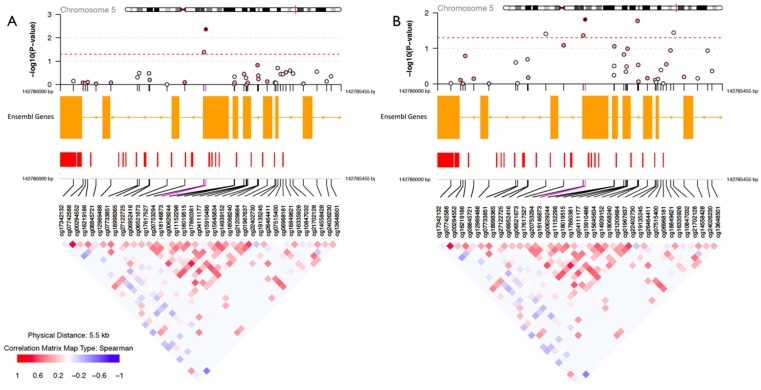
Representative regional plots illustrating part of NR3C1 gene and investigated CpGs, highlighting the differentially methylated sites in psychologically (**A**) and physically (**B**) stressed women relative to healthy women, respectively, at Time 1. The upper panels present the log transformed *p*-values with red dotted line as the cutoff used (*p* ≤ 0.05). Below is the ENSEMBL track annotation and the correlation of methylation levels among all CpGs in this selected region.

In Figure 3, we present CpG sites for each significant gene by its position and t-value before FDR correction, with hypermethylation indicated in red and hypomethylation in blue for each stress group at both time points. Both physical and psychological stress were associated with hypomethylation at two consecutive CpG sites on *NR3C1* (cg18146873 and cg20753294; map position 142782791 and 142782827) located in exon 1C of the proximal promoter [29] at T1 and one CpG site at T2 located to the promoter (cg22402730; map position 142784168). A representative regional plot of *NR3C1* gene highlighting the two consecutive CpG sites’ location at T1 and the correlation of sites within this region is shown in Figure 4. Correlation analysis shows high positive correlation among the CpG sites surrounding the two reported sites. Similarly, stress was associated with hypomethylation at two CpG sites on *FKBP5*, one CpG at T1 (cg07485685; map position 35696061), and another site at T2 (cg00130530; map position35657202). A detailed summary of the differentially methylated CpGs in candidate genes of physically and psychologically stressed participants compared to the healthy group at Time 1 and 2 are presented in Supplementary Tables S1 and S2. We report the main effect of time and the intraclass correlation (ICC) of the mixed effect model in Supplementary Table S3. Further, we performed an in-silico interaction network analysis of the eight genes of interest using the String database. This analysis highlights the close relationship of the selected genes at the level of protein interactions (Supplementary Figure S1).

## 3. Discussion

In this study, we examined DNAm on eight candidate genes associated with stress in a sample of pregnant Latina women. We did not find any significant differences in methylation of stress genes after FDR adjustment in NR3C1 and FKBP5 genes at the two different time points examined. Before adjustment, we found two sites that were hypomethylated in NR3C1 in stressed women at T1; these are located in exon 1C in the proximal promoter of the gene. Methylation differences in NR3C1 have been reported in CpG islands located in its proximal promoter in response to trauma, PTSD, depression, and anxiety [24,29] Methylation levels in NR3C1 proximal promoter is negatively associated with transcript levels [30,31,32,33], which could be associated with sensitization of the HPA axis in cases of hypomethylation. This is suggested from studies reporting hypomethylation of NR3C1 in PTSD patients where augmented feedback inhibition is observed after dexamethasone challenge [34]. Interestingly, hypomethylation in the cg20753294 site has been previously reported in blood of postpartum women exposed to war trauma [19], suggesting the relevance of the differential methylation of this site in perinatal cohorts in the context of stress exposure.

Glucocorticoid receptor (GR), encoded by the NR3C1, activity is regulated by FKBP5, which binds to GR and inhibits its translocation into the nucleus by modulating GR sensitivity to cortisol, thus acting as a short negative feedback loop [35]. Differential DNA methylation in FKBP5 gene has commonly been investigated in an intronic region spanning a GR response element and has been associated with stress and trauma [36,37]. Here, we found that stress was associated with hypomethylation of two sites in the promoter region. In a recent elegant study, hypomethylation in cg00130530 site of FKBP5 was reported to be associated with aging and stress exposure both in vivo and in vitro with functional up-regulation of FKBP5 and inflammation in blood mediated by NF-κB through a response element spanning this CpG site [38]. Despite not surviving FDR correction, our results are in line with findings in the literature in highlighting both NR3C1 and FKBP5 association with stress. While the effects of psychological stress on the epigenome have been extensively investigated, there are limited studies examining physical stress effects. Our study indicates that physical stress, like psychological stress, can leave a molecular signature in maternal blood during the prenatal period. 

Our study has several strengths. We address gaps in the literature by examining DNAm changes during two timepoints in pregnancy and include measures of physical and psychological stress combined with epigenetic markers in an all-Latina sample. Some limitations include lack of measurement of additional psychological stressors, such as racism and discrimination, that may also contribute to poor health and outcomes for Latinas, which may have resulting in residual confounding. In addition, our sample size was relatively small and composed of primarily healthy women. However, we were able to detect signals of differential methylation patterns between the stressed and healthy groups. Our findings are consistent with our hypothesis of observing differences between stressed pregnant women and healthy women and warrant further investigation and replication.

Future work should examine changes in stress induced DNAm in pregnancy and postpartum compared to preconception levels, as these have yet to be assessed in this population [39]. Knowledge of the timing of these changes may inform future screening using DNAm as a biomarker. There is also a need for a more comprehensive assessment of institutional and structural level stressors, such as racism and discrimination, that may contribute to poorer pregnancy health among some Latino subgroups. As our demographic results indicate, most Latina women in this study did not identify with White or Black race, but instead chose to identify with a native or indigenous background. Latino ethnicity includes people of different countries of origin and races and thus distinct physical phenotypes that may represent widely divergent exposures of racism and discrimination. Future studies should consider this in their study design and include samples of phenotypically divergent Latina women to interrogate adverse health outcomes of interest.

## 4. Materials and Methods

We conducted a secondary analysis of stored DNA samples and phenotype data from (n = 58) participants enrolled in the Prenatal Stress: The Epigenetic Basis of Maternal and Perinatal Effects (EPI) Study (R01MH092580, PI: Monk). Briefly, the EPI study enrolled 187 healthy participants from 2011–2016 in the late first or second trimester to observe maternal mood and stress during pregnancy [40]. Eligibility criteria included self-reported good health, English fluency, singleton gestation, and current enrollment in prenatal care. Participants were excluded from participation if they reported tobacco or drug use or use of certain psychiatric or other medications. Participants’ age ranged from 19.5–44.9 years (mean = 29.6), and 69% identified as Hispanic or Latina (n = 129). The majority reported attaining more than a high school education (mean = 14.9 years), and approximately half (49%) of participants reported Medicaid for health insurance (n = 93). Most were multiparous (n = 141). Written, informed consent was received from each participant and procedures were approved by the New York State Psychiatric Institute Institutional Review Boards.

As previously described [41], EPI participants had up to three study visits, in early (12–22 weeks; Time [T] 1), middle (23–28 weeks; T2) and late (34–36 weeks; T3) pregnancy. Maternal mood and stress groups were based on their first study visit at either T1 or T2. Several validated instruments were used to assess stress, depression, anxiety, and PTSD symptoms. Social support, biological measures (peripheral blood, height, weight, blood pressure, heart rate, and mean arterial pressure), diurnal salivary cortisol, and dietary recalls were also collected. Birth outcomes including delivery type (cesarean or vaginal birth), gestational age, complications, and birthweight were ascertained via medical record abstraction after birth. Stress phenotypes were assigned using latent profile analysis (LPA) based on the participant’s first study visit (mean = 19.7 weeks gestation, s.d. = 5.73). Three distinct groups emerged in the LPA with most participants (66.8%; n = 125) categorized into the Healthy Group and fewer assigned to the Psychologically Stressed (17.1%, n = 32) or Physically Stressed (16%, n = 30) groups. These groups were mutually exclusive and were developed by using continuous input variables to make mutually exclusive categories of stress groups. Women in the Physically Stressed group had higher mean blood pressure and mean arterial pressures, consumed significantly more calories, protein, fat, and sugar compared to those in the Healthy Group. Women in the Psychologically Stressed group had more unhappy pregnancy experiences and higher perceived stress, daily negative affect, depression, anxiety, and PTSD symptoms than women in the two other groups. Groups differed in social support levels, however, there were no differences between the three groups on maternal age, diurnal cortisol or physical activity. Physically Stressed women were significantly more likely to have a preterm birth (<37 weeks gestation) compared to those in the HG (OR = 4.07, 95% CI: 1.03–16.09) [41].

### 4.1. Methylation Analysis

Maternal T cell leukocytes were isolated from blood samples by negative selection using RosetteSep kits (Stem Cell Technologies, Vancouver, BC, Canada). To prepare specimens for methylation analysis, the 500 ng of genomic DNA was treated with bisulfite reagents using the EZ-96 DNA methylation kit (Zymo Research, Irvine, CA, USA) according to the manufacturer’s protocol. Bisulfite-converted DNA samples were then analyzed using the Illumina Infinium EPIC Methylation BeadChip array V1 [Catalog #WG-317-1003, which included the arrays and the reagents used (available from Illumina (Illumina site link https://support.illumina.com/array/array_kits/infinium-methylationepic-beadchip-kit.html) accessed on 22 June 2022)**.** This array allows for examination of more than 850,000 CpG sites at a single nucleotide resolution. EPIC analyses were conducted at the University of Minnesota Genome Center. Data pre-processing and quality control analyses were performed in R with Bioconductor package *minfi 1.40.0* and *ewastools 1.7*. Samples that failed 2 of the 17 metrics described in the BeadArray Controls Reporter Software Guide were excluded [42]. Further quality control and functional normalization were conducted as others have done [43,44]. We also removed far outliers of median methylation values and wrong sex prediction samples. No samples were removed due to wrong sex prediction. Two samples did not pass quality control, therefore we performed normal out of band background (Noob) within-sample correction, and removed probes with low intensities (detected in the *minfi 1.40.0* package) using the threshold of *p* > 0.01. Next, probe type adjustment was done using *Rcp* in the *EnMix* 1.30.0 package, and batch effects were adjusted using *combat function.* Beta (β) values for each CpG site were calculated using the *minfi 1.40.0* package. β values were defined as M/(M + U + α), where M is the total methylated signal and U is the total unmethylated signal, ranging from 0.0 to 1.0. We removed probes that were cross-hybridising using the *rmSNPandCH* module in the *DMRcate 2.8.0* package. We estimated the relative proportion of each cell type in our heterogenous peripheral blood samples [45]. We also further excluded CpG sites that were outside the 5% and 95% threshold for very low or high methylation for all samples [46]. The CpG sites were selected based on targeted genes using the EPIC array annotation file, resulting in a total of 424 CpG sites. Preprocessing excluded 15 sites leading to a final total of 409 CpG sites that were used for differential methylation analysis. Since methylation values at CpG sites can be cell-type specific [47], we conducted cell composition analysis using a modified version of Houseman and colleagues’ method [48] and the cell type proportions were adjusted using linear regression. Visualization of genomic regions with differentially methylated sites was done using the *coMET* package [49]. For preprocessing and analysis, we used R version v4.0.3 and various packages including minfi v1.40.0, ENmix 1.30.0, sva v3.42.0, DMRcate v2.8.0, and Ewastools v1.7. Also, the preprocessing script is available as the monklab.methyl package which is available in https://github.com/seonjoo/monklab.methyl accessed on 22 June 2022.

### 4.2. Stress Candidate Gene Selection

Candidate genes and CpG sites are presented in Table 1.

### 4.3. Statistical Analysis

We examined selected CpGs on the candidate genes to compare differences in methylation by stress group, as well as investigating associations within the whole sample. We used linear regression models to examine each stress group (Physically and Psychologically Stressed) compared to the Healthy Group as a predictor of methylation of each candidate stress gene. We adjusted for maternal age, fetal sex, parity, proportions of cell types of T lymphocytes (CD4+ and CD8+), B cells (CD19+), monocytes (CD14+), NK cells (CD56+) and neutrophils, and performed multiple comparisons correction controlling for false discovery rate (FDR) [50].

## Figures and Tables

**Table 1:** Sample characteristics of Latina pregnant women from the EPI Study by stress group, N = 58.

	Healthy (n = 34)		Psychologically Stressed (n = 15)		Physically Stressed (n = 9)		Total (N = 58)	
	n (Mean)	% (s.d.)	n (Mean)	% (s.d.)	n (Mean)	% (s.d.)	n (Mean)	% (s.d.)
Mean (s.d.) maternal age at enrollment (T1)								
	27.2	5.5	27.6	4.6	26.0	3.5	27.1	5.0
Race								
American Indian/Alaskan	20	58.8	8	53.3	6	66.7	34	58.6
Black/African American	1	2.9	2	13.3	3	33.3	6	10.3
White	8	23.5	3	20.0	0	0.0	11	19.0
Biracial	2	5.9	0	0.0	0	0.0	2	3.4
Other	3	8.8	2	13.3	0	0.0	3	8.6
Fetal sex								
Male	21	61.8	6	40.0	1	11.1	28	48.3
Female	13	38.2	9	60.0	8	88.9	30	51.7
Parity								
0	30	88.2	12	80.0	8	88.9	50	86.2
1	4	11.8	3	20.0	1	11.1	8	13.8
Gestational age at birth (weeks)	39.47	1.78	38.8	2.7	39.1	1.47	39.24	2.0
Highest education completed								
High School graduate/GED	22	64.7	10	71.4	8	88.9	40	70.2
Associate degree/College graduate or higher	12	35.3	4	28.6	1	11.1	17	29.8
Annual household income								
≤$15,000	6	17.6	5	33.3	1	11.1	12	20.7
>$15,000–$50,000	14	41.2	7	46.7	7	77.8	28	48.3
≥$50,000	14	41.2	3	20.0	1	11.1	18	31.0
Medicaid								
Yes	21	61.8	12	85.7	8.0	88.9	41	71.9
No	13	38.2	2	14.3	1.0	11.1	16	28.1
Current smoker								
Yes	0	0	0	0.0	0.0	0.0	0.0	0.0
No	34	100	14	100.0	9.0	100.0	57	100.0
Ever smoker								
Yes	9	26.5	6	42.9	4	44.4	19	33.3
No	25	73.5	8	57.1	5	55.6	38	66.7

**Table 2:** Candidate stress genes and CpG sites.

Gene	Number of CpGs
*BDNF*	90
*CRHR1*	40
*CRHR2*	40
*FKBP5*	50
*HSD11B2*	22
*NR3C1*	88
*NR3C2*	49
*SLC6A4*	30
Total	409

## Data Availability

Data from this study are available from the authors upon reasonable request. The genomic dataset is currently in submission to GEO.

## Supplementary Materials

The following supporting information can be downloaded at: https://www.mdpi.com/article/10.3390/epigenomes7040027/s1, Table S1: Differentially methylated CpGs in candidate genes of physically and psychologically stressed women relative to healthy women at T1 (12–22 weeks); Table S2: Differentially methylated CpGs in candidate genes of physically and psychologically stressed women relative to healthy women at T2 (23–28 weeks); Table S3: Consistency of methylation between the two time points. We provide intraclass correlation (ICC) of the mixed effect model including only time as the main effect adjusting for covariates. The CpG sites were ordered by the magnitude of ICC; Figure S1: Network analysis and plot of the eight genes of interest performed in-silico through the String database.
