# Supplementary material for: Stress and DNA Methylation of Blood Leukocytes among Pregnant Latina Women
Source: Epigenomes. 2023 Nov 1;7(4):27. doi: 10.3390/epigenomes7040027 (PMC10660842; doi:10.3390/epigenomes7040027)
Supplement: Supplementary file 1 [file epigenomes-07-00027-s001.zip › epigenomes-2627254-supplementary.pdf]

Table S1. Differentially methylated CpGs in candidate genes of physically and psychologically stressed women relative to healthy women at T1 (12-22 weeks)

CpG methylated region	Gene	Cohen's d	beta	Standard error	Unadjusted p-value	FDR corrected p-value	Average Methylation (%) *	CHR	Base pair position	Relation to CpG island	Regulatory Feature Group	UCSC RefGene Group
Physically stressed vs. Healthy women												
cg13157799	*NR3C2*	1.3286	0.048	0.0137	0.0015	0.62	11.57%	chr4	149191991	Open Sea		Body
cg07633853	*FKBP5*	-1.1894	-0.039	0.0125	0.0039	0.7327	13.37%	chr6	35569471	Open Sea	Unclassified Cell type	Body
cg05877083	*CRHR2*	-1.1407	-0.037	0.0121	0.0054	0.7327	85.15%	chr7	30703457	Open Sea		Body
cg02712145	*CRHR2*	-0.9785	-0.030	0.0116	0.0151	0.8529	7.68%	chr7	30722433	Island	Unclassified	TSS1500
cg09160032	*NR3C2*	0.9764	0.011	0.0042	0.0153	0.8529	94.08%	chr4	149347020	Open Sea		Body
cg18146873	*NR3C1*	-0.9751	-0.007	0.0027	0.0154	0.8529	2.84%	chr5	142782827	Island		1stExon;5'UTR
cg14939152	*NR3C1*	-0.9605	-0.004	0.0014	0.0169	0.8529	2.34%	chr5	142783831	Island	Promoter Associated	5'UTR;TSS1500
cg09251165	*CRHR1*	0.9239	0.014	0.0055	0.0211	0.8529	92.76%	chr17	43894089	Open Sea		5'UTR;Body
cg02527472	*BDNF*	-0.9146	-0.026	0.0108	0.0223	0.8529	10.13%	chr11	27743348	N Shore		TSS1500; 1stExon; 5'UTR

cg26949694	*BDNF*	0.9128	0.022	0.0092	0.0225	0.8529	23.70%	chr11	27742060	Island	Unclassified Cell type	Body; 5’UTR; TSS1500; 1stExon
cg01583131	*BDNF*	0.8514	0.038	0.0169	0.0323	0.8529	23.38%	chr11	27744675	S Shore		TSS1500
cg06046431	*BDNF*	-0.8361	- 0.004	0.0016	0.0353	0.8529	3.67%	chr11	27744490	Island		TSS1500
cg10847032	*NR3C1*	0.8321	0.005	0.002	0.0361	0.8529	1.94%	chr5	142784522	Island	Promoter Associated	TSS1500;5’UTR
cg08388004	*BDNF*	0.8291	0.015	0.0068	0.0367	0.8529	92.48%	chr11	27679632	Open Sea		Body;1stExon
cg06521673	*NR3C1*	0.8186	0.004	0.0018	0.039	0.8529	1.69%	chr5	142782072	Island		5’UTR
cg08636224	*FKBP5*	0.8143	0.005	0.0024	0.04	0.8529	95.71%	chr6	35657921	S Shore		5’UTR;TSS1500
cg07485685	*FKBP5*	-0.8079	- 0.016	0.0072	0.0414	0.8529	6.43%	chr6	35696061	Island	Promoter Associated	5’UTR;Body
cg15462887	*BDNF*	0.8062	0.023	0.0106	0.0418	0.8529	7.40%	chr11	27744049	Island		TSS1500
cg20753294	*NR3C1*	-0.7997	- 0.016	0.0076	0.0434	0.8529	7.68%	chr5	142782791	Island		1stExon;5’UTR
cg16012111	*FKBP5*	0.7881	0.016	0.0075	0.0463	0.8529	4.20%	chr6	35656758	Island	Promoter Associated	TSS200;5’UTR
cg25381667	*BDNF*	-0.7852	- 0.016	0.0075	0.0471	0.8529	9.18%	chr11	27743651	Island		TSS200;TSS1500
cg21010859	*BDNF*	-0.7834	- 0.017	0.0081	0.0475	0.8529	4.33%	chr11	27740161	N Shore		Body;5’UTR
cg01913022	*CRHR2*	0.7817	0.066	0.0317	0.048	0.8529	41.67%	chr7	30740409	Open Sea		TSS1500

Psychologically stressed vs. Healthy Women

cg24249411	*BDNF*	1.3832	0.040	0.011	0.001	0.4247	11.10%	chr11	27744759	S Shore		TSS1500
cg18146873	*NR3C1*	-1.1731	-0.009	0.0027	0.0043	0.8872	2.84%	chr5	142782827	Island		1stExon;5’UTR
cg02122296	*CRHR1*	-0.9879	-0.003	0.0013	0.0143	0.9981	1.50%	chr17	43909656	Open Sea	Unclassified	Body
cg07485685	*FKBP5*	-0.9745	-0.019	0.0072	0.0155	0.9981	6.43%	chr6	35696061	Island	Promoter Associated	5’UTR;Body
cg14297797	*CRHR1*	0.9712	0.019	0.0072	0.0158	0.9981	92.44%	chr17	43867801	Open Sea		5’UTR;Body
cg06937024	*FKBP5*	0.8846	0.007	0.0029	0.0266	0.9981	1.97%	chr6	35695489	N Shore	Promoter Associated	5’UTR;Body
cg01991100	*SLC6A4*	-0.8723	-0.042	0.018	0.0286	0.9981	73.22%	chr17	28555935	Open Sea		5’UTR
cg16692923	*NR3C2*	-0.8696	-0.008	0.0033	0.0291	0.9981	2.61%	chr4	149364700	Island		TSS1500
cg07704699	*BDNF*	0.8548	0.061	0.0271	0.0317	0.9981	18.36%	chr11	27742832	N Shore	Unclassified Cell type	Body;TSS1500
cg12081455	*HSD11B2*	0.8537	0.026	0.0116	0.0319	0.9981	34.03%	chr16	67465561	S Shore		Body
cg20753294	*NR3C1*	-0.8093	-0.016	0.0076	0.0411	0.9981	7.68%	chr5	142782791	Island		1stExon;5’UTR
cg12790440	*HSD11B2*	0.8063	0.028	0.0132	0.0418	0.9981	8.52%	chr16	67464868	Island	Unclassified Cell type	TSS200

			-									
cg04856689	*CRHR1*	-0.7981	0.008	0.0036	0.0438	0.9981	4.52%	chr17	43862032	Island		Body
cg13094036	*CRHR2*	0.7787	0.002	0.0008	0.0488	0.9981	1.37%	chr7	30722359	Island	Unclassified Cell type	TSS1500

* Average methylation was computed as the mean of the 1st trimester methylation data (n=40).

Table S2. Differentially methylated CpGs in candidate genes of physically and psychologically stressed women relative to healthy women at T2 (23-28 weeks)

CpG methylated region	Gene	Cohen's d	beta	Standard error	Unadjusted p-value	FDR corrected P-value	Average Methylation (%)*	CHR	Base pair position	Relation to CpG island	Regulatory Feature Group	UCSC RefGene Group
Physically stressed vs. Healthy Women												
cg07778819	*CRHR1*	0.8296	0.032	0.0129	0.0161	0.9964	14%	chr17	43862927	Island		Body
cg00130530	*FKBP5*	-0.8129	-0.050	0.0202	0.0181	0.9964	75%	chr6	35657202	S Shore	Promoter Associated	5'UTR; TSS1500
cg01819552	*CRHR2*	0.7156	0.031	0.014	0.036	0.9964	72%	chr7	30706917	Open Sea		Body
cg13648501	*NR3C1*	-0.6874	-0.007	0.0034	0.0435	0.9964	5%	chr5	142785258	S Shore	Promoter Associated	
cg22402730	*NR3C1*	-0.6841	-0.002	0.0012	0.0444	0.9964	2%	chr5	142784168	Island	Promoter Associated	5'UTR; TSS1500

Psychologically stressed vs. Healthy Women

cg08695103	*NR3C1*	-0.8936	-0.029	0.0107	0.0099	0.9976	18%	chr5	142733619	Open Sea		Body
cg22402730	*NR3C1*	-0.7808	-0.002	0.001	0.0229	0.9976	2%	chr5	142784168	Island	Promoter Associated	5’UTR; TSS1500
cg19645279	*NR3C1*	-0.7675	-0.033	0.0141	0.0251	0.9976	55%	chr5	142702733	Open Sea		Body
cg08473090	*CRHR1*	-0.7285	-0.004	0.002	0.0329	0.9976	4%	chr17	43861104	Island		TSS1500
cg01972879	*CRHR2*	0.7044	0.025	0.0117	0.0388	0.9976	85%	chr7	30720901	N Shore		Body
cg00130530	*FKBP5*	-0.6806	-0.035	0.0167	0.0455	0.9976	75%	chr6	35657202	S Shore	Promoter Associated	5’UTR; TSS1500
cg07651033	*NR3C2*	0.6791	0.004	0.0019	0.0459	0.9976	95%	chr4	149359043	Open Sea		5’UTR

* Average methylation was computed as the mean of the 2nd trimester methylation data (n=49).

Table S3. Consistency of methylation between the two time points. We provide intraclass correlation (ICC) of the mixed effect model including only time as the main effect adjusting for covariates. The CpG sites were ordered by the magnitude of ICC.

		Main Effect of Time					
CpG	ICC	Numerator DF	Denominator DF	F-value	P-value	Adjusted p-value	Gene
cg18090898	0.848	1	35.049	0.166	0.687	0.966	CRHR2
cg06175988	0.828	1	29.304	0.017	0.898	0.994	CRHR2
cg11524343	0.811	1	36.88	1.533	0.223	0.723	CRHR1;MGC57346-CRHR1
cg03484834	0.79	1	40.643	4.138	0.048	0.586	CRHR2
cg16912838	0.761	1	35.182	0.563	0.458	0.872	FKBP5
cg03323388	0.76	1	31.379	0.378	0.543	0.916	CRHR1;MGC57346-CRHR1
cg17924854	0.749	1	39.202	3.364	0.074	0.586	CRHR2
cg16647683	0.732	1	29.929	2.869	0.101	0.612	SLC6A4
cg04106006	0.714	1	39.395	0.014	0.905	0.994	BDNF
cg12296752	0.699	1	39.072	2.203	0.146	0.667	BDNF;BDNF-AS
cg12511160	0.692	1	41.99	6.835	0.012	0.586	CRHR2
cg24052866	0.682	1	39.3	0.81	0.374	0.835	NR3C1
cg27107893	0.681	1	35.976	0.149	0.702	0.966	NR3C1
cg16052510	0.643	1	40.149	0	0.997	0.997	FKBP5
cg17617527	0.642	1	40.497	0.907	0.347	0.801	NR3C1

cg10635145	0.639	1	41.362	0.136	0.714	0.966	BDNF
cg03943825	0.635	1	40.902	0.956	0.334	0.783	SLC6A4
cg07704699	0.626	1	42.074	3.846	0.057	0.586	BDNF
cg11015767	0.622	1	36.247	0.05	0.825	0.985	NR3C2
cg25563198	0.622	1	40.673	0.317	0.576	0.923	FKBP5;LOC285847
cg04863452	0.605	1	36.097	0.336	0.566	0.923	CRHR2
cg07657976	0.594	1	32.177	0.235	0.631	0.952	CRHR1;MGC57346-CRHR1
cg08760147	0.59	1	41.145	1.524	0.224	0.723	BDNF;BDNF-AS
cg13094036	0.589	1	33.043	0.708	0.406	0.848	CRHR2
cg26656751	0.583	1	39.858	0.049	0.826	0.985	CRHR1
cg02613510	0.572	1	42.427	0.223	0.64	0.954	BDNF
cg27605489	0.571	1	36.037	1.486	0.231	0.723	CRHR2
cg01819552	0.571	1	42.136	0.442	0.51	0.909	CRHR2
cg07733851	0.567	1	31.535	0.251	0.62	0.939	NR3C1
cg01546433	0.567	1	39.53	0.108	0.744	0.972	BDNF
cg19176661	0.561	1	42.775	0.638	0.429	0.852	NR3C1
cg22043168	0.56	1	40.516	3.825	0.057	0.586	BDNF
cg12969488	0.551	1	29.894	0.078	0.781	0.985	NR3C1
cg19457823	0.546	1	39.354	6.513	0.015	0.586	NR3C1
cg06087101	0.539	1	39.186	0.136	0.714	0.966	FKBP5
cg23430507	0.531	1	39.48	0.137	0.713	0.966	NR3C1

cg19645279	0.528	1	37.527	0.154	0.697	0.966	NR3C1
cg05877083	0.513	1	41.855	6.899	0.012	0.586	CRHR2
cg20728768	0.509	1	37.4	0.54	0.467	0.872	NR3C1
cg14896516	0.502	1	38.806	0.286	0.596	0.927	CRHR2
cg05951817	0.5	1	42.678	1.868	0.179	0.716	SLC6A4
cg01049782	0.496	1	35.15	0.262	0.612	0.937	CRHR2
cg13344434	0.495	1	36.903	0.013	0.91	0.994	FKBP5
cg00052684	0.494	1	39.487	1.289	0.263	0.728	FKBP5
cg08818984	0.494	1	44.403	0.638	0.429	0.852	NR3C1
cg03667083	0.491	1	42.539	0.035	0.853	0.985	CRHR2
cg05189570	0.491	1	42.922	0.118	0.733	0.972	BDNF;BDNFOS
cg16224829	0.488	1	38.999	1.449	0.236	0.723	NR3C1
cg27460943	0.483	1	46.455	1.404	0.242	0.723	NR3C2
cg24394631	0.478	1	45.811	0.137	0.713	0.966	CRHR1
cg13678281	0.477	1	44.042	2.002	0.164	0.699	HSD11B2
cg16642545	0.475	1	38.369	2.719	0.107	0.612	CRHR1
cg25928860	0.475	1	37.184	0.049	0.826	0.985	BDNF;BDNF-AS
cg01967637	0.469	1	37.585	0.634	0.431	0.852	NR3C1
cg10022526	0.468	1	48.272	0.255	0.616	0.938	BDNF
cg17342132	0.468	1	42.7	0.109	0.743	0.972	NR3C1
cg26035844	0.467	1	41.9	0.021	0.885	0.994	NR3C2

cg08845721	0.466	1	42.779	3.181	0.082	0.586	NR3C1
cg05366813	0.465	1	40.592	1.54	0.222	0.723	CRHR2
cg18351440	0.46	1	42.809	0.047	0.829	0.985	CRHR2
cg17349736	0.457	1	40.393	5.359	0.026	0.586	NR3C1
cg02386994	0.456	1	41.527	7.379	0.01	0.586	BDNF;BDNF-AS
cg27304079	0.451	1	45.282	0.505	0.481	0.878	NR3C2
cg18911683	0.451	1	43.513	0.332	0.567	0.923	CRHR2
cg26262196	0.45	1	37.974	0.844	0.364	0.823	CRHR2
cg03066966	0.448	1	42.057	1.034	0.315	0.77	CRHR1;MGC57346-CRHR1
cg14297797	0.447	1	39.704	0.046	0.831	0.985	CRHR1;MGC57346-CRHR1
cg24353392	0.443	1	37.551	0.002	0.965	0.994	CRHR1
cg06866646	0.443	1	38.802	0.002	0.965	0.994	CRHR2
cg14438279	0.442	1	36.18	0.312	0.58	0.923	NR3C1
cg04867484	0.441	1	39.951	0.05	0.824	0.985	NR3C2
cg25639595	0.438	1	42.327	0.117	0.734	0.972	CRHR2
cg14642437	0.438	1	35.587	3.18	0.083	0.586	FKBP5
cg17085721	0.437	1	33.58	1.749	0.195	0.723	FKBP5
cg22584138	0.433	1	45.8	0.656	0.422	0.852	SLC6A4
cg16219186	0.43	1	43.995	0	0.997	0.997	NR3C1
cg18019515	0.429	1	41.954	0.137	0.713	0.966	NR3C1
cg12067298	0.426	1	37.868	1.258	0.269	0.728	BDNF

cg03245912	0.425	1	43.439	0.194	0.662	0.959	FKBP5
cg11731737	0.424	1	27.431	1.459	0.237	0.723	CRHR1
cg05437692	0.416	1	42.173	0.112	0.74	0.972	NR3C2
cg27234800	0.416	1	39.442	1.64	0.208	0.723	NR3C2
cg14939152	0.416	1	41.361	0.396	0.533	0.916	NR3C1
cg08362738	0.415	1	44.547	1.735	0.194	0.723	BDNF
cg16535116	0.414	1	40.743	6.134	0.018	0.586	NR3C1
cg13974632	0.411	1	45.855	0.002	0.961	0.994	BDNF
cg18998365	0.402	1	41.244	0.071	0.791	0.985	NR3C1
cg25114611	0.399	1	44.269	1.642	0.207	0.723	FKBP5;LOC285847
cg13947929	0.398	1	36.103	0.119	0.732	0.972	CRHR1
cg22233604	0.397	1	44.317	0.127	0.723	0.972	NR3C1
cg16755766	0.394	1	43.145	0.04	0.842	0.985	CRHR2
cg11905112	0.387	1	39.563	2.434	0.127	0.644	FKBP5
cg07515400	0.386	1	47.091	1.13	0.293	0.746	NR3C1
cg24295963	0.382	1	36.569	0.589	0.448	0.872	FKBP5
cg24063856	0.38	1	46.854	0.408	0.526	0.916	CRHR1
cg08059229	0.38	1	42.539	1.009	0.321	0.777	NR3C2
cg00022871	0.379	1	40.204	1.235	0.273	0.728	CRHR1
cg15690037	0.379	1	43.804	1.557	0.219	0.723	HSD11B2
cg25328597	0.375	1	38.86	5.213	0.028	0.586	BDNF

cg11806762	0.374	1	41.256	5.59	0.023	0.586	BDNF
cg01225698	0.372	1	42.287	3.708	0.061	0.586	BDNF
cg04457787	0.362	1	42.901	3.524	0.067	0.586	NR3C1
cg16692923	0.359	1	48.519	0.001	0.976	0.994	NR3C2
cg04481212	0.358	1	46.488	1.325	0.256	0.728	BDNF
cg09251165	0.352	1	45.687	0.905	0.346	0.801	CRHR1;MGC57346-CRHR1
cg15115787	0.351	1	39.366	0.166	0.686	0.966	NR3C1
cg07760722	0.351	1	38.173	3.114	0.086	0.586	NR3C2
cg01443318	0.346	1	47.71	1.103	0.299	0.746	HSD11B2
cg00386645	0.34	1	45.137	1.837	0.182	0.716	SLC6A4
cg24270678	0.34	1	42.45	1.133	0.293	0.746	HSD11B2
cg12081455	0.34	1	42.983	1.333	0.255	0.728	HSD11B2
cg19432243	0.338	1	39.589	4.085	0.05	0.586	NR3C1
cg27191795	0.338	1	41.389	0.003	0.957	0.994	CRHR2
cg25579735	0.335	1	45.526	0.65	0.424	0.852	NR3C1
cg24984698	0.334	1	47.828	0.001	0.977	0.994	SLC6A4
cg23185751	0.333	1	43.826	0.988	0.326	0.783	CRHR2
cg04923928	0.329	1	46.438	0.037	0.849	0.985	CRHR2
cg20954537	0.327	1	45.166	0.37	0.546	0.916	BDNF
cg06991510	0.327	1	46.043	0.09	0.765	0.984	BDNF
cg18718518	0.326	1	40.29	0.004	0.948	0.994	NR3C1

cg04137760	0.326	1	44.68	3.586	0.065	0.586	FKBP5
cg13373360	0.326	1	38.411	0.007	0.932	0.994	NR3C2
cg16586394	0.324	1	42.193	0.559	0.459	0.872	NR3C1
cg20813374	0.322	1	44.243	1.363	0.249	0.728	FKBP5
cg12670061	0.321	1	46.274	0.001	0.969	0.994	HSD11B2
cg21291635	0.319	1	35.897	0.324	0.573	0.923	BDNF
cg06409316	0.318	1	46.86	4.289	0.044	0.586	FKBP5
cg05733135	0.318	1	43.652	2.655	0.11	0.612	BDNF
cg24249411	0.317	1	39.02	0.022	0.884	0.994	BDNF
cg02534661	0.317	1	41.482	0.443	0.509	0.909	NR3C2
cg11580341	0.315	1	40.49	0.653	0.424	0.852	NR3C2
cg26949694	0.312	1	43.691	0.052	0.82	0.985	BDNF
cg03546163	0.312	1	43.792	0.012	0.912	0.994	FKBP5
cg15607306	0.312	1	41.342	0.393	0.534	0.916	CRHR1
cg04750517	0.309	1	45.177	0.778	0.383	0.835	HSD11B2
cg20981893	0.309	1	48.76	0.059	0.809	0.985	HSD11B2
cg01751279	0.308	1	47.041	0.024	0.876	0.994	NR3C1
cg27410679	0.307	1	49.507	0.054	0.817	0.985	CRHR1
cg26840770	0.306	1	48.135	2.755	0.103	0.612	BDNF
cg16005389	0.306	1	44.589	3.09	0.086	0.586	FKBP5
cg14291693	0.301	1	47.729	0.253	0.617	0.938	BDNF;BDNFOS

cg06979684	0.297	1	46.427	4.02	0.051	0.586	BDNF;BDNFOS
cg09238384	0.296	1	45.202	1.696	0.199	0.723	NR3C2
cg26464411	0.293	1	49.046	0.56	0.458	0.872	NR3C1
cg12074493	0.293	1	48.11	1.74	0.193	0.723	SLC6A4
cg23947039	0.29	1	38.546	0.044	0.836	0.985	BDNF
cg15929276	0.286	1	38.561	0.544	0.465	0.872	FKBP5
cg08264907	0.282	1	46.578	5.465	0.024	0.586	NR3C2
cg15645634	0.279	1	44.529	0.02	0.888	0.994	NR3C1
cg05483455	0.279	1	44.167	5.255	0.027	0.586	NR3C1
cg00407401	0.279	1	44.853	4.966	0.031	0.586	NR3C1
cg13157799	0.278	1	41.512	0.007	0.935	0.994	NR3C2
cg24026230	0.277	1	47.388	0.092	0.763	0.984	NR3C1
cg19491599	0.277	1	38.551	2.16	0.15	0.667	NR3C2
cg03906910	0.275	1	50.279	0.291	0.592	0.927	NR3C1
cg07696519	0.272	1	43.127	2.525	0.119	0.64	FKBP5
cg08636224	0.27	1	39.029	0.104	0.748	0.975	FKBP5
cg27427014	0.269	1	44.707	0.055	0.816	0.985	SLC6A4
cg09505801	0.268	1	47.882	0.314	0.578	0.923	BDNF
cg11241206	0.265	1	47.963	0.369	0.546	0.916	BDNF
cg27551605	0.264	1	41.985	0.124	0.727	0.972	CRHR1
cg02810898	0.263	1	48.294	0.019	0.891	0.994	CRHR1;MGC57346-CRHR1

cg23329208	0.263	1	44.501	1.114	0.297	0.746	NR3C2
cg16335926	0.261	1	44.187	1.259	0.268	0.728	NR3C1
cg25672354	0.259	1	47.441	0.011	0.918	0.994	NR3C2
cg18117895	0.259	1	48.635	1.253	0.268	0.728	BDNF
cg20598211	0.258	1	47.345	3.307	0.075	0.586	NR3C1
cg23776787	0.257	1	38.914	0.378	0.542	0.916	NR3C1
cg09422970	0.253	1	46.893	2.191	0.145	0.667	CRHR1;MGC57346-CRHR1
cg25156688	0.252	1	51.06	0.286	0.595	0.927	BDNF
cg17311440	0.252	1	50.464	0.035	0.852	0.985	CRHR1;MGC57346-CRHR1
cg27503360	0.25	1	47.734	1.977	0.166	0.701	CRHR1
cg14284211	0.25	1	48.227	0.188	0.666	0.959	FKBP5
cg00328411	0.25	1	46.219	1.771	0.19	0.723	NR3C2
cg04111177	0.249	1	37.232	0.072	0.789	0.985	NR3C1
cg24525872	0.248	1	45.104	0.487	0.489	0.885	NR3C2
cg24801588	0.243	1	46.422	4.427	0.041	0.586	NR3C1
cg24065044	0.241	1	45.645	2.733	0.105	0.612	BDNF
cg18484679	0.238	1	33.076	0	0.991	0.996	NR3C1
cg12466613	0.237	1	40.812	2.119	0.153	0.667	NR3C1
cg16545496	0.237	1	43.439	3.065	0.087	0.586	HSD11B2
cg07715663	0.236	1	45.442	5.371	0.025	0.586	NR3C1
cg22128379	0.234	1	41.851	1.235	0.273	0.728	BDNF

cg21783716	0.233	1	41.931	2.118	0.153	0.667	NR3C2
cg08929103	0.232	1	37.757	0.208	0.651	0.958	CRHR1
cg05900547	0.229	1	43.858	5.095	0.029	0.586	NR3C1
cg24214442	0.229	1	46.7	4.383	0.042	0.586	CRHR2
cg00629244	0.226	1	48.422	1.518	0.224	0.723	NR3C1
cg03984780	0.225	1	42.999	1.06	0.309	0.761	BDNF
cg11760414	0.224	1	49.466	0.017	0.898	0.994	CRHR1;MGC57346-CRHR1
cg24650785	0.219	1	46.667	0.004	0.95	0.994	BDNF
cg11338426	0.218	1	36.637	0.362	0.551	0.916	CRHR1
cg08423118	0.217	1	45.453	1.444	0.236	0.723	NR3C1
cg22363520	0.216	1	44.978	0.019	0.89	0.994	FKBP5
cg15014679	0.215	1	46.739	2.643	0.111	0.612	BDNF;BDNFOS
cg06669759	0.215	1	36.881	0.471	0.497	0.895	NR3C2
cg25412831	0.214	1	45.239	0.541	0.466	0.872	BDNF
cg19014730	0.213	1	42.857	0.662	0.42	0.852	FKBP5
cg12946179	0.212	1	50.033	0.723	0.399	0.848	NR3C2
cg27193031	0.211	1	41.3	0.023	0.88	0.994	BDNF
cg10993059	0.209	1	50.004	2.341	0.132	0.644	NR3C2
cg07778819	0.208	1	47.166	0.001	0.976	0.994	CRHR1
cg09268536	0.208	1	41.75	2.782	0.103	0.612	FKBP5
cg24430106	0.208	1	50.547	2.527	0.118	0.64	CRHR2

cg20556751	0.205	1	38.195	0.153	0.698	0.966	HSD11B2
cg12888360	0.204	1	41.511	2.678	0.109	0.612	NR3C1
cg01972879	0.204	1	50.687	0.294	0.59	0.927	CRHR2
cg17860381	0.204	1	44.257	5.8	0.02	0.586	NR3C1
cg19226017	0.203	1	48.58	3.027	0.088	0.586	FKBP5;LOC285847
cg12741214	0.195	1	50.179	1.362	0.249	0.728	NR3C1
cg20209182	0.194	1	36.859	0.077	0.784	0.985	SLC6A4
cg00294552	0.192	1	47.776	7.549	0.008	0.586	NR3C1
cg01718447	0.192	1	43.774	1.93	0.172	0.706	CRHR2
cg02665568	0.192	1	45.989	12.293	0.001	0.42	FKBP5
cg12448003	0.191	1	49.831	0.324	0.572	0.923	BDNF
cg13000004	0.191	1	44.15	3.698	0.061	0.586	NR3C2
cg15688670	0.188	1	44.03	0.216	0.644	0.954	BDNF
cg14621978	0.185	1	42.544	3.029	0.089	0.586	NR3C1
cg12790440	0.184	1	44.916	0.019	0.89	0.994	HSD11B2
cg23497217	0.183	1	36.456	1.416	0.242	0.723	BDNF
cg07485685	0.183	1	42.581	0.722	0.4	0.848	FKBP5;LOC285847
cg10256584	0.18	1	37.672	0.289	0.594	0.927	CRHR1;MGC57346-CRHR1
cg03746860	0.18	1	47.474	1.604	0.211	0.723	NR3C1
cg01642653	0.179	1	45.86	0.949	0.335	0.783	BDNF
cg02122296	0.178	1	47.321	3.096	0.085	0.586	CRHR1;MGC57346-CRHR1

cg25708981	0.176	1	44.45	2.998	0.09	0.586	NR3C1
cg01991100	0.176	1	48.978	2.384	0.129	0.644	SLC6A4
cg18068240	0.175	1	51.464	0.053	0.819	0.985	NR3C1
cg18266052	0.17	1	48.174	0.769	0.385	0.835	CRHR2
cg18584905	0.169	1	50.36	0.186	0.668	0.959	SLC6A4
cg26081259	0.166	1	44.417	7.841	0.008	0.586	NR3C1
cg23426002	0.162	1	39.647	0.762	0.388	0.835	BDNF;BDNFOS
cg01913022	0.162	1	47.717	4.184	0.046	0.586	CRHR2
cg03829016	0.16	1	31.743	0.441	0.511	0.909	SLC6A4
cg07335874	0.159	1	43.71	0.786	0.38	0.835	NR3C2
cg04922810	0.158	1	42.128	4.668	0.036	0.586	CRHR2
cg20340655	0.154	1	41.986	2.322	0.135	0.647	BDNF
cg12841684	0.153	1	43.478	1.351	0.251	0.728	NR3C2
cg06843189	0.15	1	52.659	0.003	0.958	0.994	NR3C2
cg10300814	0.15	1	34.316	0.768	0.387	0.835	FKBP5
cg04672351	0.15	1	34.592	0.558	0.46	0.872	BDNF
cg06046431	0.149	1	39.762	1.846	0.182	0.716	BDNF
cg07742588	0.148	1	45.511	2.026	0.161	0.695	NR3C1
cg25381667	0.148	1	48.443	0.113	0.738	0.972	BDNF
cg23273257	0.147	1	43.221	2.434	0.126	0.644	NR3C1
cg25535999	0.147	1	48.253	0.003	0.958	0.994	NR3C1

cg07589972	0.143	1	48.972	3.539	0.066	0.586	NR3C1
cg07238832	0.143	1	43.56	0.657	0.422	0.852	BDNF;BDNFOS
cg14312898	0.141	1	51.006	0.043	0.837	0.985	SLC6A4
cg08743901	0.141	1	43.188	0.88	0.353	0.806	SLC6A4
cg26495008	0.14	1	46.046	3.802	0.057	0.586	FKBP5
cg21773872	0.138	1	54.515	0.001	0.982	0.995	CRHR2
cg19820298	0.136	1	36.782	2.156	0.151	0.667	NR3C1
cg10901968	0.136	1	49.906	0.073	0.788	0.985	SLC6A4
cg06240648	0.133	1	47.45	1.916	0.173	0.706	NR3C2
cg10146136	0.13	1	48.111	0.761	0.387	0.835	SLC6A4
cg18595174	0.129	1	34.734	0.398	0.532	0.916	BDNF
cg06613263	0.128	1	30.748	1.46	0.236	0.723	NR3C1
cg27430726	0.125	1	43.424	1.431	0.238	0.723	CRHR2
cg13514002	0.12	1	46.436	0.39	0.535	0.916	NR3C1
cg15117716	0.12	1	39.894	0.201	0.656	0.958	CRHR1;MGC57346-CRHR1
cg10207656	0.12	1	49.457	0.499	0.483	0.879	NR3C2
cg07843056	0.119	1	44.035	1.635	0.208	0.723	FKBP5
cg18534039	0.119	1	49.424	1.302	0.259	0.728	CRHR1
cg00140191	0.118	1	52.53	0.102	0.751	0.975	FKBP5
cg07919246	0.118	1	45.798	0.319	0.575	0.923	BDNF;BDNF-AS
cg05016953	0.117	1	46.977	1.902	0.174	0.706	SLC6A4

cg13521908	0.117	1	42.288	0.505	0.481	0.878	CRHR1
cg24396090	0.117	1	48.439	0.034	0.855	0.985	NR3C2
cg00130530	0.116	1	37.491	0.012	0.913	0.994	FKBP5
cg06260077	0.113	1	47.667	1.492	0.228	0.723	BDNF
cg11865360	0.113	1	47.914	0.979	0.327	0.783	BDNF
cg21701890	0.113	1	45.035	3.265	0.077	0.586	NR3C2
cg12021170	0.113	1	50.717	4.732	0.034	0.586	BDNF;BDNF-AS
cg11845071	0.112	1	49.966	2.295	0.136	0.647	FKBP5;LOC285847
cg24610236	0.111	1	47.306	1.209	0.277	0.728	CRHR2
cg08043197	0.111	1	54.988	2.789	0.101	0.612	CRHR1;MGC57346-CRHR1
cg25962210	0.111	1	47.619	0.874	0.355	0.806	BDNF
cg07633853	0.11	1	51.646	2.803	0.1	0.612	FKBP5
cg17882499	0.107	1	50.602	0.1	0.754	0.975	BDNF
cg07545640	0.105	1	52.938	0.171	0.681	0.966	HSD11B2
cg17413943	0.1	1	49.416	0.209	0.649	0.958	BDNF
cg21702128	0.1	1	8.033	2.819	0.132	0.644	NR3C1
cg13753571	0.097	1	33.296	0.359	0.553	0.916	HSD11B2
cg16012111	0.087	1	53.954	0.41	0.525	0.916	FKBP5
cg06816235	0.085	1	54.251	0.526	0.471	0.872	BDNF
cg01330016	0.084	1	47.992	0.338	0.564	0.923	SLC6A4
cg10241426	0.082	1	45.012	0.424	0.518	0.916	SLC6A4

cg15615793	0.08	1	28.301	5.024	0.033	0.586	CRHR2
cg08711598	0.08	1	49.112	4.576	0.037	0.586	HSD11B2
cg01294526	0.079	1	47.247	0.041	0.841	0.985	NR3C1
cg10847032	0.078	1	45.592	1.207	0.278	0.728	NR3C1
cg09143276	0.075	1	48.331	1.156	0.288	0.744	NR3C2
cg09921370	0.075	1	43.465	0.529	0.471	0.872	SLC6A4
cg23420656	0.073	1	48.402	0.219	0.642	0.954	CRHR1;MGC57346-CRHR1
cg27345592	0.069	1	46.902	0.822	0.369	0.829	NR3C1
cg03167496	0.068	1	51.455	0.004	0.947	0.994	BDNF
cg01516788	0.065	1	47.429	1.098	0.3	0.746	NR3C2
cg10106856	0.064	1	46.694	0.188	0.666	0.959	CRHR1;MGC57346-CRHR1
cg20108357	0.062	1	39.786	0.008	0.928	0.994	BDNF
cg02955911	0.061	1	49.596	0.358	0.552	0.916	HSD11B2
cg15313332	0.058	1	47.722	3.956	0.052	0.586	BDNF
cg00862770	0.055	1	51.071	1.327	0.255	0.728	FKBP5
cg22826063	0.055	1	48.176	0.643	0.427	0.852	CRHR2
cg10590842	0.055	1	53.44	0.003	0.96	0.994	NR3C2
cg09516959	0.051	1	50.232	3.179	0.081	0.586	CRHR2
cg15910486	0.048	1	40.104	0.038	0.847	0.985	NR3C1
cg18146873	0.047	1	49.212	0.085	0.771	0.985	NR3C1
cg26054404	0.047	1	50.5	1.28	0.263	0.728	NR3C2

cg27225476	0.045	1	47.122	2.17	0.147	0.667	NR3C2
cg27122725	0.044	1	49.088	3.477	0.068	0.586	NR3C1
cg17253842	0.04	1	51.29	0.054	0.818	0.985	NR3C2
cg02734600	0.039	1	54.152	1.556	0.218	0.723	HSD11B2
cg21789597	0.038	1	45.147	0.204	0.654	0.958	MIR5690;FKBP5
cg08789908	0.036	1	51.179	0.963	0.331	0.783	HSD11B2
cg05075176	0.036	1	51.533	1.296	0.26	0.728	NR3C2
cg26741280	0.035	1	49.853	3.145	0.082	0.586	SLC6A4
cg16127724	0.033	1	50.514	3.913	0.053	0.586	CRHR2
cg22046703	0.032	1	49.299	0.508	0.479	0.878	CRHR1;MGC57346-CRHR1
cg07275757	0.03	1	47.012	1.737	0.194	0.723	NR3C2
cg06968181	0.029	1	52.143	0.138	0.712	0.966	NR3C1
cg05039098	0.027	1	32.145	3.765	0.061	0.586	FKBP5
cg06684850	0.027	1	45.817	1.963	0.168	0.701	BDNF
cg14589148	0.021	1	49.812	1.408	0.241	0.723	BDNF
cg20140452	0.02	1	55.096	3.043	0.087	0.586	NR3C2
cg03857453	0.018	1	50.505	0.173	0.68	0.966	NR3C1
cg16594263	0.018	1	46.311	2.399	0.128	0.644	NR3C1
cg11718030	0.016	1	41.501	3.705	0.061	0.586	BDNF
cg21010859	0.014	1	55.577	0	0.988	0.995	BDNF
cg25725890	0.012	1	45.483	0.357	0.553	0.916	SLC6A4

cg01294490	0.011	1	36.929	0	0.984	0.995	FKBP5
cg09606766	0.011	1	52.413	0.01	0.921	0.994	BDNF
cg04791658	0.008	1	48.398	1.558	0.218	0.723	FKBP5
cg21209684	0.007	1	41.748	1.215	0.277	0.728	NR3C1
cg22288103	0.004	1	50.976	0.008	0.931	0.994	BDNF
cg08473090	0	1	42.05	0.22	0.641	0.954	CRHR1
cg09160032	0	1	78	0.043	0.835	0.985	NR3C2
cg19650300	0	1	78	1.594	0.211	0.723	NR3C2
cg07651033	0	1	78	0.154	0.696	0.966	NR3C2
cg01576854	0	1	78	0.403	0.528	0.916	NR3C2
cg10288772	0	1	78	0.014	0.906	0.994	NR3C2
cg04424630	0	1	78	0.602	0.44	0.862	NR3C2
cg22391185	0	1	78	1.551	0.217	0.723	NR3C2
cg08695103	0	1	78	3.106	0.082	0.586	NR3C1
cg06952416	0	1	78	0.614	0.436	0.857	NR3C1
cg06521673	0	1	78	0.018	0.895	0.994	NR3C1
cg20753294	0	1	78	0.004	0.952	0.994	NR3C1
cg11152298	0	1	78	0.007	0.933	0.994	NR3C1
cg22402730	0	1	78	0.26	0.612	0.937	NR3C1
cg19135245	0	1	78	3.058	0.084	0.586	NR3C1
cg18849621	0	1	78	1.591	0.211	0.723	NR3C1

cg14558428	0	1	78	0.396	0.531	0.916	NR3C1
cg13648501	0	1	78	1.425	0.236	0.723	NR3C1
cg13764763	0	1	78	0.763	0.385	0.835	NR3C1
cg26720913	0	1	78	2.444	0.122	0.64	NR3C1
cg21979215	0	1	78	1.154	0.286	0.744	NR3C1
cg01839003	0	1	78	0.22	0.64	0.954	FKBP5
cg07061368	0	1	78	2.941	0.09	0.586	FKBP5
cg03591753	0	1	78	0.718	0.399	0.848	FKBP5
cg08642543	0	1	78	0.681	0.412	0.852	FKBP5
cg03098337	0	1	78	1.018	0.316	0.77	FKBP5
cg14339974	0	1	78	0.955	0.332	0.783	FKBP5
cg23416081	0	1	78	7.031	0.01	0.586	FKBP5
cg06937024	0	1	78	0.142	0.707	0.966	FKBP5;LOC285847
cg00610228	0	1	78	0.044	0.834	0.985	FKBP5;LOC285847
cg17030679	0	1	78	0.164	0.687	0.966	FKBP5;LOC285847
cg07658503	0	1	78	0.032	0.859	0.987	CRHR2
cg02712145	0	1	78	0.709	0.402	0.848	CRHR2
cg19920989	0	1	78	5.153	0.026	0.586	CRHR2
cg08388004	0	1	78	2.079	0.153	0.667	BDNF;BDNFOS
cg23143371	0	1	78	0.134	0.715	0.966	BDNF;BDNF-AS
cg09492354	0	1	78	5.52	0.021	0.586	BDNF

cg26057780	0	1	78	0.027	0.87	0.994	BDNF
cg10558494	0	1	78	0.011	0.916	0.994	BDNF
cg23619332	0	1	78	0.67	0.415	0.852	BDNF
cg00298481	0	1	78	0.004	0.95	0.994	BDNF
cg07159484	0	1	78	0.337	0.563	0.923	BDNF
cg06025631	0	1	78	0.05	0.823	0.985	BDNF
cg15710245	0	1	78	0.002	0.961	0.994	BDNF
cg03747251	0	1	78	1.097	0.298	0.746	BDNF
cg15914769	0	1	78	0.699	0.406	0.848	BDNF
cg05218375	0	1	78	0.14	0.709	0.966	BDNF
cg24377657	0	1	78	0.084	0.773	0.985	BDNF
cg01636003	0	1	78	0.037	0.848	0.985	BDNF
cg05818894	0	1	78	0.525	0.471	0.872	BDNF
cg27351358	0	1	78	0.11	0.741	0.972	BDNF
cg02527472	0	1	78	0.035	0.851	0.985	BDNF
cg16257091	0	1	78	0.049	0.826	0.985	BDNF
cg25457956	0	1	78	0.26	0.612	0.937	BDNF
cg15462887	0	1	78	0.197	0.658	0.958	BDNF
cg01583131	0	1	78	2.841	0.096	0.612	BDNF
cg27130954	0	1	78	0.867	0.355	0.806	HSD11B2
cg02322203	0	1	78	0.014	0.905	0.994	HSD11B2

cg10686375	0	1	78	0.038	0.845	0.985	HSD11B2
cg07724674	0	1	78	1.648	0.203	0.723	HSD11B2
cg09807841	0	1	78	0.562	0.456	0.872	HSD11B2
cg20592995	0	1	78	1.195	0.278	0.728	SLC6A4
cg06961290	0	1	78	0.085	0.771	0.985	SLC6A4
cg26067340	0	1	78	0.039	0.843	0.985	SLC6A4
cg26126367	0	1	78	0.287	0.594	0.927	SLC6A4
cg14692377	0	1	78	0.003	0.958	0.994	SLC6A4
cg26438554	0	1	78	0.278	0.599	0.928	SLC6A4
cg06373684	0	1	78	2.459	0.121	0.64	SLC6A4
cg06841846	0	1	78	1.75	0.19	0.723	SLC6A4
cg12577105	0	1	78	0.002	0.968	0.994	CRHR1
cg18757974	0	1	78	5.168	0.026	0.586	CRHR1
cg04856689	0	1	78	0.012	0.914	0.994	CRHR1
cg24738082	0	1	78	1.084	0.301	0.746	CRHR1;MGC57346-CRHR1
cg16830379	0	1	78	0	0.987	0.995	CRHR1

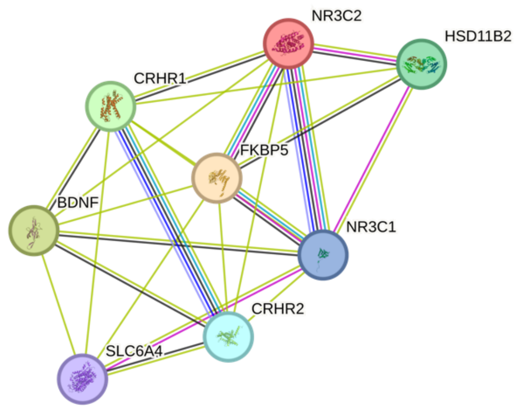

Figure S1. Network analysis and plot of the eight genes of interest performed in-silico through the String database. Link to the full analysis: https://string-db.org/cgi/network?taskId=balcng8LZOvh&sessionId=brRDbug88f8s
